# ALDH1A1 expression correlates with clinicopathologic features and poor prognosis of breast cancer patients: a systematic review and meta-analysis

**DOI:** 10.1186/1471-2407-14-444

**Published:** 2014-06-17

**Authors:** Ying Liu, Dong-lai Lv, Jiang-jie Duan, Sen-lin Xu, Jing-fang Zhang, Xiao-jun Yang, Xia Zhang, You-hong Cui, Xiu-wu Bian, Shi-cang Yu

**Affiliations:** 1 Institute of Pathology and Southwest Cancer Center, Southwest Hospital, Third Military Medical University, Chongqing 400037, China; 2 Key Laboratory of Tumor Immunology and Pathology of Ministry of Education, Chongqing 400037, China; 3 School of Biomedical Sciences, The Chinese University of Hong Kong, Hongkong, China

**Keywords:** Breast cancer, Mammary cancer, Cancer stem cell, Aldehyde dehydrogenase 1 family member A1, Prognosis

## Abstract

**Background:**

Aldehyde dehydrogenase 1 family member A1 (ALDH1A1) has been identified as a putative cancer stem cell (CSC) marker in breast cancer. However, the clinicopathological and prognostic significance of this protein in breast cancer patients remains controversial.

**Methods:**

This meta-analysis was conducted to address the above issues using 15 publications covering 921 ALDH1A1^+^ cases and 2353 controls. The overall and subcategory analyses were performed to detect the association between ALDH1A1 expression and clinicopathological/prognostic parameters in breast cancer patients.

**Results:**

The overall analysis showed that higher expression of ALDH1A1 is associated with larger tumor size, higher histological grade, greater possibility of lymph node metastasis (LNM), higher level expression of epidermal growth factor receptor 2 (HER2), and lower level expression of estrogen receptor (ER)/progesterone receptor (PR). The prognosis of breast cancer patients with ALDH1A1^+^ tumors was poorer than that of the ALDH1A1^-^ patients. Although the relationships between ALDH1A1 expression and some clinicopathological parameters (tumor size, LNM, and the expression of HER2) was not definitive to some degree when we performed a subcategory analysis, the predictive values of ALDH1A1 expression for histological grade and survival of breast cancer patients were significant regardless of the different cutoff values of ALDH1A1 expression, the different districts where the patients were located, the different clinical stages of the patients, the difference in antibodies used in the studies, and the surgery status.

**Conclusions:**

Our results indicate that ALDH1A1 is a biomarker to predict tumor progression and poor survival of breast cancer patients. This marker should be taken into consideration in the development of new diagnostic and therapeutic program for breast cancer.

## Background

Cancer stem cells (CSCs), although being a small percentage of the cancer cell population, are characterized by their multipotency and the ability to initiate cancer and propagate metastases [1-3]. Since the first report of these cells, which were found among acute myeloid leukemia cells by cell sorting technology using multiple surface markers [4], CSCs have been reported in various tumors, such as colon cancer [5], brain tumor [6], and lung cancer [7]. Due to their high tumorigenic and metastatic potential, CSCs are thought to be the most formidable obstacle to the successful treatment of cancer.

CSCs also have been isolated from breast cancer [8,9], the most common malignancy in women worldwide. In 2003, Al-Hajj *et al*. have identified and isolated breast CSCs from patients using the cell surface marker pattern CD44^+^CD24^-/low^Lineage^-^[10]. Subsequently, Ginestier *et al*. have reported that the activity of aldehyde dehydrogenase 1 (ALDH1) as assessed by the Aldefluor assay is a specific indicator for identifying, isolating, and tracking human breast CSCs [11].

The ALDH1A subfamily comprises three isoforms (ALDH1A1, ALDH1A2, and ALDH1A3), which synthesize retinoic acid (RA) from the retina and are crucial regulators for the RA signaling pathway. These enzymes have a high affinity for the oxidation of both all-trans- and 9-cis-retinal and thereby serve to regulate the self-renewal and differentiation of normal stem cells and CSCs [12].

Although the exact isoform of ALDH1A responsible for the enzymatic activity assessed by BODIPY aminoacetaldehyde remains controversial [13-16], aldehyde dehydrogenase 1 family member A1 (ALDH1A1) is thought to have a predominant role [17]. Thus, much attention has been focused on the relationship between the expression of this isoform and the clinicopathologic parameters, including prognosis, of breast cancer patients.

However, the prognostic value of ALDH1A1 for breast cancer remains controversial despite numerous independent studies. For example, in a series of 577 breast carcinomas, Christophe Ginestier *et al*. demonstrated that ALDH1A1 expression detected by immunostaining correlated with poor patient prognosis [11]. Mieog *et al*. have revealed that the prognostic value of ALDH1A1 expression is age dependent and can be observed only in patients aged < 65 years [18]. Using a retrospective collection of 321 node-negative and 318 node-positive breast cancer patients with a mean follow-up time of 12.6 years, Neumeister *et al*. found that ALDH1A1 expression alone does not significantly predict therapeutic outcome [19]. Therefore, we performed a systematic review and a meta-analysis to assess the robustness of the relationship between ALDH1A1 expression and clinicopathologic parameters/outcomes in breast cancer patients.

## Methods

### Search strategy

We conducted a search of the PubMed and EMBASE databases to identify studies for the systematic review. Two major groups of studies were created according to our objective. One group was used to clarify the association between ALDH1A1 expression and clinicopathological parameters, including tumor size, lymph node metastasis (LNM), histological grade, and the expression of growth factor receptors (estrogen receptor, ER; progesterone receptor, PR; epidermal growth factor receptor 2, HER2). The other group was used to investigate the association between ALDH1A1 expression and overall survival (OS)/disease-free survival (DFS).

The search terms were “ALDH1”, “breast cancer”. All studies were published prior to March 13, 2014. In the initial retrieved literatures, we read the titles or abstracts and screened for prognosis- and clinicopathology-related research. Studies were included when the following criteria were met: (1) published in English with the full text available, (2) the use of a case control design or a cohort design, and (3) the availability of data to allow the estimation of the hazard ratio (HR) for survival with a 95% CI. Accordingly, the exclusion criteria were as follows: (1) reviews, abstracts and repeated studies; (2) ALDH1A1 not specified as the subtype expressed; and (3) the use of duplicate data. No ethnicity or regional restrictions were applied. The review process was performed by two independent reviewers.

### Data extraction

The following information was extracted from these papers based the criteria listed above: first author, patients' country, publication year, research technique used, number of cases and controls, cutoff value for ALDH1A1, antibody used, type of tumor samples, and HR. For references that did not provide HRs, we referred to the methods described by Tierney *et al*. [20] to obtain the HRs using the data and figures from the original papers [19,21-23].

### Statistical analysis

The prognosis of patients with breast cancer positive for ALDH1A1 expression was calculated using the unadjusted HR with the corresponding 95% CI according the OS/specific survival (SS)/relative survival (RS) and DFS/metastasis-free survival (MFS)/recurrence-free survival (RFS) in cases and controls. We classified different prognostic parameters from included references, based on the characteristics of censored data, into two groups: (1) OS/SS/RS; (2) DFS/MFS/RFS. Other clinicopathological factors were sorted into several subgroups: tumor size, LNM, histological grade, and the expression of ER, PR, and HER2. Fixed and random effects models were used to calculate a pooled odds ratio (OR) and HR. The statistical significance of the pooled OR and HR was evaluated with the Z test and P values, and *P* < 0.05 was considered statistically significant. Heterogeneity across studies was evaluated by applying a Q test. In this approach, the Q value is defined as identical to the effect size of the *I*^*2*^ value. A random effects model was used when the *I*^*2*^ value for heterogeneity test was >50%; otherwise, a fixed effects model was used. Begg’s rank correlation method and Egger’s weighted regression method were used to assess publication bias (*P* < 0.05 was considered statistically significant). All statistical tests for this meta-analysis were performed using STATA 11.0 software (STATA Corp., College Station, TX, USA).

## Results

### Study characteristics

**Figure 1:**
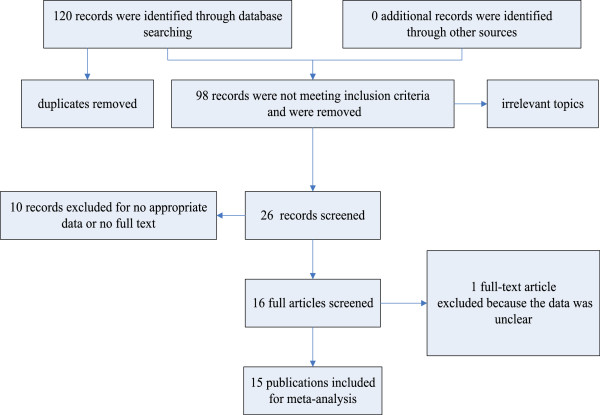
Flow chart of eligible study identification.

A total of 16 studies from 15 publications [11,18,19,21-32] were found to meet the criteria for this analysis after the article titles, abstracts and main text were read to identify case reports and clinical outcomes. The flow chart for the identification of eligible studies is shown in Figure 1. The total number of patients was 3274, including 921 cases ALDH1A1^+^ breast cancer and a 2353 controls. Except in the study by Neumeister, immunohistochemistry (IHC) was a primary method used to evaluate ALDH1A1 expression in breast cancer specimens [19]. We identified the detected subtype as ALDH1A1 based on the antibodies listed in the references. For uniformed data analysis, tumor size T1 was considered as low stage, and T2, T3, and T4 as high stage. For the histological grade, all the studies used Nottingham Combined Histology Grade modified Scarff-Bloom-Richardson (SBR) grading system, grades I and II were grouped together *vs*. grade III. In the study by Ginestier *et al*., the patient samples were derived from two independent groups (America and France) [11]. Therefore, these samples were divided into two studies: the Ginestier U.M. set and the Ginestier I.P.C. set. The prognostic data from Lee *et al*. [26] was not available, because it was evaluated according to the change of expression of ALDH1A1 before and after the chemotherapy, rather than the categories ALDH1A1^+^ and ALDH1A1^-^. The main characteristics of the 16 eligible studies are summarized in Table 1.

**Table 1:** Main characteristics of the eligible studies

**Author**	**Country**	**Year**	**Method**	**Cases**	**Controls**	**Cutoff of**	**Dilution of antibody**	**Situation of patients**	**HR**
				**(ALDH1A1**^**+**^**)**	**(ALDH1A1**^**-**^**)**	**ALDH1A1 positive**			
Ginestier U.M. [11]	America	2007	IHC (TMA)	24	122	> 5%	BD Biosciences, 1:100	NA	NA
Ginestier I.P.C [11]	France	2007	IHC (TMA)	102	243	> 5%	BD Biosciences, 1:100	NA	1.76
Morimoto [27]	Japan	2009	IHC	21	182	> 0%	BD Biosciences, 1:100	Treated with surgery	1.516
Charafe-Jauffret [24]	France	2010	IHC	29	53	> 1%	BD Biosciences, 1:50	IBC, partly treated with surgery	SS, 2.7
									MFS, 2.72
Erika Resetkova [29]	America	2010	IHC (TMA)	35	159	> 0%	BD Biosciences, 1:200	Treated with surgery	NA
Nalwoga [28]	Uganda	2010	IHC (TMA)	88	95	unclear	BD Biosciences, 1:250	NA	NA
Neumeister [19]	America	2010	Immunofluorescent assays (AQUA)	45	581	NA	BD Biosciences, 1:1000	Treated with surgery	2.32
Pei Yu [23]	China	2010	IHC	18	78	> 0%	Abcam, 1:100	Treated with surgery	4.6
He Lee [26]	Korea	2011	IHC	12	80	>5%	BD Biosciences, 1:100	Stage II ~ III, treated with surgery	4.15
Yasuyo [31]	Japan	2011	IHC	54	52	> 0%	BD Biosciences, 1:1000	TNBC, treated with surgery	3.696
Yoshioka [32]	Japan	2011	IHC	68	189	> 0%	BD Biosciences, 1:1000	Treated with surgery	OS, 1.93
									RFS, 1.667
Mieog [18]	Netherlands	2012	IHC	292	195	> 0%	BD Biosciences, NA	Treated with surgery	RS, 2.36
									RFS, 1.71
Nogami [21]	Japan	2012	IHC	7	33	> 5%	BD Biosciences, 1:200	ALNM^+^, treated with surgery	2.26
Sakakibara [22]	Japan	2012	IHC	35	80	>5%	BD Biosciences, 1:200	ALNM^+^, treated with surgery	10.044
Tan [30]	China, Malay, Indian, Other	2013	IHC	35	106	>10%	Abcam, 1:100	Treated with surgery	NA
Dong [25]	China	2013	IHC	56	105	>5%	BD Biosciences, 1:200	Invasive ductal carcinoma and ALNM^+^, treated with surgery	OS, 3.309
									RFS, 2.774

a) IBC was defined as inflammatory breast cancer, it is stage IIIB.

b) TMA was defined as tissue microarrays.

c) AQUA was defined as automated quantitative analysis.

d) TNBC breast cancer was defined as triple-negative breast cancer.

e) ALNM was defined as axillary lymph node metastases, it is ≥ Stage II.

### Meta-analysis results

#### Correlation of ALDH1A1 expression with clinicopathological parameters

##### Overall analysis

**Figure 2:**
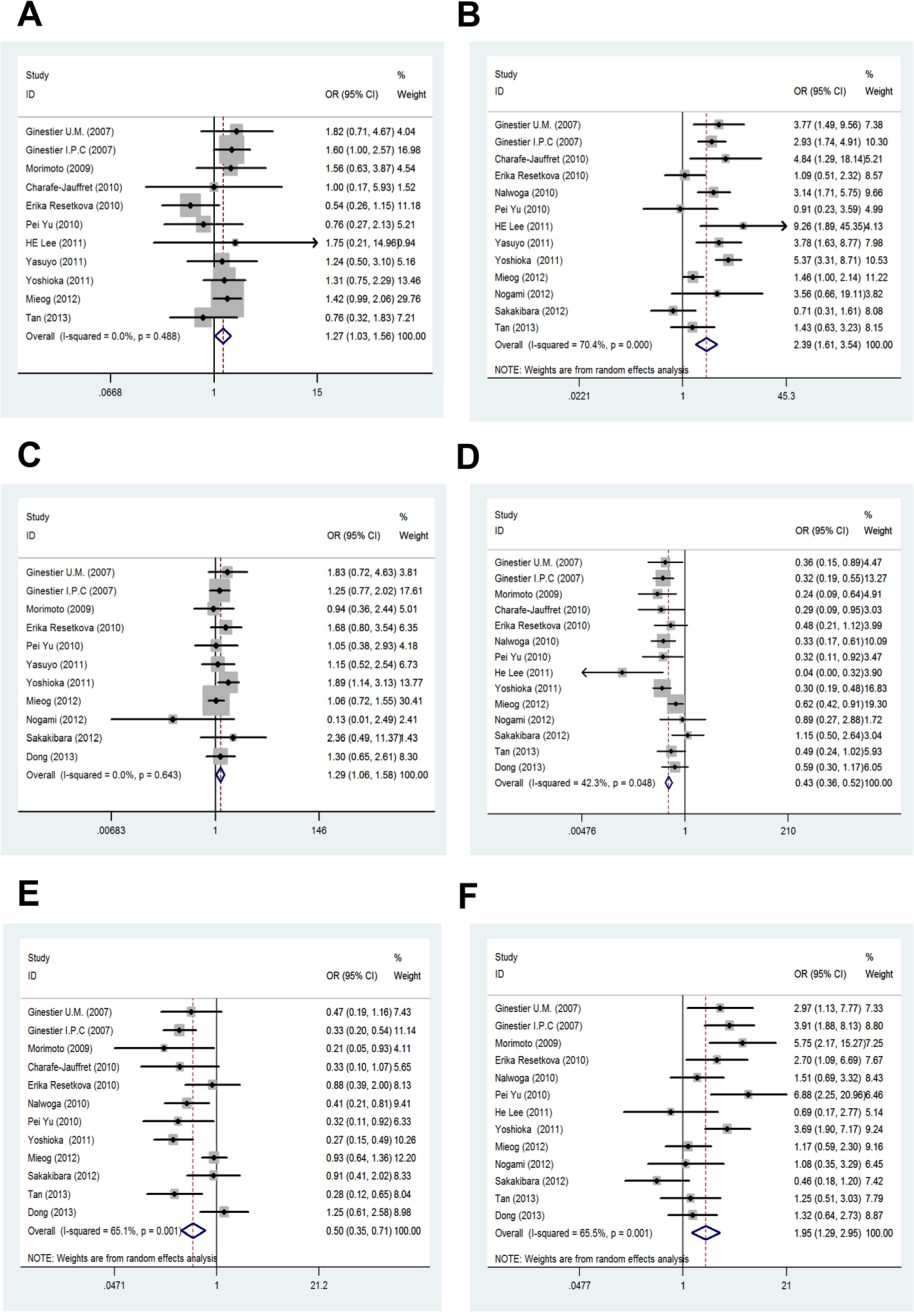
Meta-analysis of the association between ALDH1A1 expression and clinicopathological parameters: (A) LNM; (B) histological grade; (C) tumor size; (D) the expression of ER; (E) the expression of PR; (F) the expression of HER2.

There were 14 references [11,18,21-32] that assessed ALDH1A1 expression and correlated it to tumor clinicopathological data. The overall analysis showed significant association between ALDH1A1 expression and tumor size, histological grade, LNM, and the expression of ER, PR, and HER2. Specifically, higher ALDH1A1 expression means greater tumor size, higher SBR grade, greater possibility of LNM, higher expression of HER2, and lower expression of ER and PR. The results are shown in Figure 2 and Table 2.

**Table 2:** Main results of meta-analysis according to the different cutoff values of ALDH1A1expression

**Parameter**	**Cutoff value**				**Patients’ district**				**Patients’ stage**			
	**5%**		**0% or 1%**		**America-Europe**		**Asia**		**NA**		**≥Stage II**	
	**OR/HR**	**95% CI**	**OR/HR**	**95% CI**	**OR/HR**	**95% CI**	**OR/HR**	**95% CI**	**OR/HR**	**95% CI**	**OR/HR**	**95% CI**
Tumor size	1.30	0.92-1.83	1.29*	1.01-1.65	1.24	0.95-1.61	1.37*	1.01-1.86	1.31*	1.06-1.62	1.19	0.66-2.14
LNM	1.43	0.99-2.07	1.20	0.94-1.54	1.33*	1.03-1.72	1.16	0.82-1.64	1.27*	1.03-1.56	1.29	0.33-4.97
SBR grade	2.33*	1.22-4.43	2.32*	1.20-4.49	2.16*	1.31-3.55	2.41*	1.16-5.04	2.37*	1.57-3.58	2.94	0.80-10.77
ER	0.46*	0.34-0.61	0.43*	0.33-0.55	0.47*	0.36-0.61	0.42*	0.32-0.54	0.41*	0.33-0.50	0.54*	0.36-0.82
PR	0.54*	0.30-0.98	0.46*	0.25-0.84	0.56*	0.33-0.96	0.45*	0.24-.0.84	0.43*	0.29-0.64	0.82	0.42-1.61
HER2	1.39	0.78-2.48	3.19*	1.67-6.08	2.39*	1.33-4.28	1.78	0.93-3.42	2.66*	1.76-4.03	0.89	0.54-1.48
OS/SS/RS	2.65*	1.82-3.86	2.33*	1.60-3.38	2.39*	1.83-3.13	3.10*	2.02-4.75	2.25*	1.71-2.95	3.51*	2.33-5.31
DFS/MFS/RFS	2.65*	1.54-4.57	2.04*	1.53-2.73	1.95*	1.33-2.85	2.36*	1.67-3.32	1.93*	1.41-2.65	2.68*	1.73-4.13
**Parameter**	**Antibodies used in studies**				**Surgery situation of patients**				**Overall**			
	**BD**		**Abcam**		**Surgery**		**NS/Partly**					
	**OR/HR**	**95% CI**	**OR/HR**	**95% CI**	**OR/HR**	**95% CI**	**OR/HR**	**95% CI**	**OR/HR**	**95% CI**	***P***	***I***^***2***^
Tumor size	1.30*	1.06-1.60	1.05	0.38-2.93	-	-	-	-	1.29*	1.06-1.58	0.012	0.0%
LNM	1.34*	1.08-1.67	0.76	0.39-1.48	-	-	-	-	1.27*	1.03-1.56	0.024	0.0%
SBR grade	2.65*	1.73-4.08	1.27	0.63-2.56	-	-	-	-	2.39*	1.61-3.54	0.000	70.4%
ER	0.43*	0.35-0.52	0.43*	0.36-0.52	-	-	-	-	0.43*	0.36-0.52	0.000	42.3%
PR	0.54*	0.36-0.80	0.30*	0.15-0.57	-	-	-	-	0.50*	0.35-0.71	0.000	65.1%
HER2	1.85*	1.19-2.87	2.83	0.53-15.07	-	-	-	-	1.95*	1.29-2.95	0.002	65.5%
OS/SS/RS	-	-	-	-	2.87*	2.17-3.80	1.76*	1.06-2.91-	2.58*	2.05-3.23	0.000	38.0%
DFS/MFS/RFS	2.07*	1.60-2.69	4.60*	1.53-13.81	2.09*	1.60-2.75	2.72*	1.32-5.60	2.16*	1.68-2.79	0.000	0.0%

Ps. *means a significant difference.

##### Subcategory analysis

Subsequently, we performed a subcategory analysis according to different cutoff values of ALDH1A1 expression (>5% and >0%/1% subgroups), different regions the patients originated from (America-Europe, Asia, and Africa subgroups), different clinical stages of the patients [No assesment (NA) and ≥ stage II subgroups], different antibodies used in the studies (BD subgroup and Abcam subgroup), and types of surgery for patients [Surgery, Part surgery, and No screened (NS) subgroups].

In the subgroup analysis based on the cutoff value, we found that ALDH1A1 expression is positively correlated with histological grade and negatively correlated with the expression of ER/PR, which is consistent with the results derived from overall analysis. At the same time, greater tumor size and higher expression of HER2 in the ALDH1A1 positive group could be found in the subgroup studies with cutoff values >0% or 1%. However, LNM status is not correlated with ALDH1A1 expression regardless of cutoff value (Table 2 and Additional file 1: Figure S1).

Because there was only one study for African patients, meta-analysis was performed for the America-Europe and Asia subcategories according to different regions of the patients. We found that the relationship between ALDH1A1 expression and histological grade or the expression of ER/PR is the same as the results from previous overall analysis, regardless of regions of origin. However, tumor size in the America-Europe subgroup is not related to ALDH1A1 expression. In addition, greater possibility of LNM and higher expression of HER2 could be found in America-Europe patients with high ALDH1A1 expression in tumor (Table 2 and Additional file 2: Figure S2).

For subcategory analysis based on the clinical stage, six clinicopathological parameters are all correlated with ALDH1A1 expression in the NA group. However, in the group ≥ stage II, ALDH1A1 expression is only correlated with ER expression (Table 2 and Additional file 3: Figure S3).

For subcategory analysis based on the antibodies, six clinicopathological parameters are also correlated with ALDH1A1 expression in the BD group. In the Abcam group, ALDH1A1 expression is only correlated with the expression of ER and PR (Table 2 and Additional file 4: Figure S4).

#### Impact of ALDH1A1 expression on survival for breast cancer

There were a total of 11 references [11,18,19,21-25,27,31,32] relating to the association between ALDH1A1 expression and breast cancer prognosis. The prognosis was evaluated by the indicators OS/SS/RS and DFS/MFS/RFS. The studies by Charafe-Jauffret [24], Yoshioka [32] and Mieog [18] used two types of prognosis indicators, which were classified by characteristics; OS/SS/RS made up one group, DFS/MFS/RFS made up the other group.

##### Overall analysis

**Figure 3:**
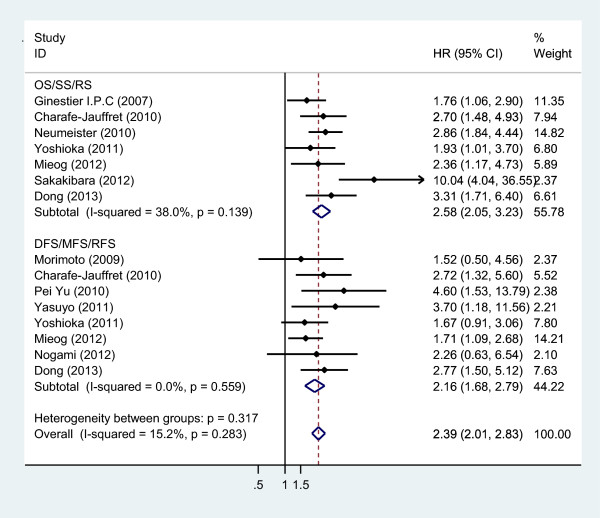
Meta-analysis of the association between ALDH1A1 expression and prognosis, including OS/SS/RS and DFS/MFS/RFS.

The data for this analysis indicated that the prognosis of breast cancer patients with ALDH1A1^+^ was poorer than that of the ALDH1A1^-^ patients regardless of the indicators used (OS/SS/RS or DFS/MFS/RFS). The results were shown as follows: OS/SS/RS: OR = 2.58, 95% CI = 2.05–3.23, *P* = 0.000, *I*^*2*^ = 38.0%; DFS/MFS/RFS: OR = 2.16, 95% CI = 1.68–2.79, *P* = 0.000, *I*^*2*^ = 0.0% (Figure 3).

##### Subcategory analysis

ALDH1A1^+^ breast cancer patients have poorer prognosis in all subcategory analysis. The results are shown in Table 2, Figure 3 and Additional file 5: Figures S5, Additional file 6: Figure S6, Additional file 7: Figure S7, Additional file 8: Figure S8 and Additional file 9: Figure S9.

##### Sensitivity analysis

Sensitivity analysis was performed through the sequential omission of individual studies. The corresponding pooled OR was not altered significantly for any study factor after sequentially excluding each study, demonstrating that our data are stable and reliable.

### Publication bias

**Figure 4:**
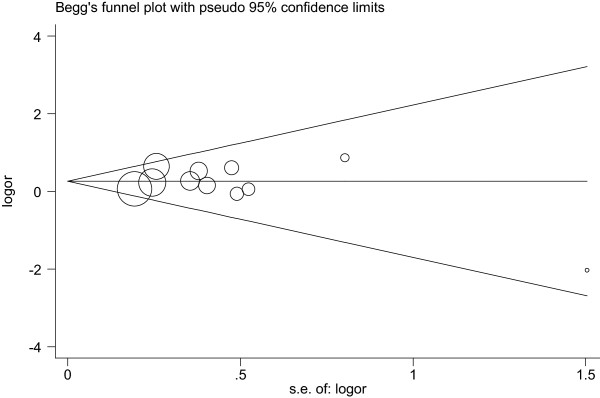
**Begg’s funnel plot of publication bias.** Each point represents a separate study for the indicated association (Tumor size).

Begg’s funnel plot and Egger’s test were used to evaluate the publication bias of all the relevant literature. The statistical results did not show evidence of publication bias: tumor size: Begg’s test, *P* = 0.755, Egger’s test, *P* = 0.721; LNM: Begg’s test, *P* = 0.640, Egger’s test, *P* = 0.342; histological grade: Begg’s test, *P* = 0.583, Egger’s test, *P* = 0.766; expression of ER: Begg’s test, *P* = 0.511, Egger’s test, *P* = 0.360; expression of PR: Begg’s test, *P* = 0.537, Egger’s test, *P* = 0.278; expression of HER2: Begg’s test, *P* = 0.855, Egger’s test, *P* = 0.749. Similar results were found for OS/SS/RS: Begg’s test, *P* = 0.368, Egger’s test, *P* = 0.155; DFS/MFS/RFS: Begg’s test, *P* = 0.266, Egger’s test, *P* = 0.169. The funnel plot used to investigate the relationship between ALDH1A1 expression and tumor size is shown in Figure 4. The shape of the funnel plot did not show obvious evidence of asymmetry.

## Discussion

It is well known that ALDH1A1 can be used as a marker for breast CSCs, which have high tumor-initiating and self-renewal capabilities. Because of the important role performed by breast CSCs in tumorigenesis, development, and therapeutic outcomes, many groups have investigated the relationship between the expression of ALDH1A1 and the clinicopathologic features of breast cancer patients. However, there are discrepancies among the studies attempted to assess the association. Our results derived from the meta-analysis of existing studies indicated that ALDH1A1 can be used as a poor prognostic indicator in breast cancer patients. The high expression of ALDH1A1 is positively associated with larger tumor size, higher histological grade and a greater likelihood of LNM in breast cancer patients. In addition, the expression of ALDH1A1 was positively correlated with the expression of HER2 but negatively correlated with the expression of ER/PR. Moreover, if we performed subcategory analysis based on the different cutoff values of ALDH1A1 expression, the different regions of origin of the patients, the different clinical stages of the patients selected, and the different antibodies used in studies, the relationships between ALDH1A1 expression and some clinicopathological parameters, including tumor size, LNM, and the expression of HER2, are slightly different. For example, the positive correlation between ALDH1A1 expression and the tumor size only could be found in the cutoff >0/1%, Asia, NA, and BD subgroups. Regarding LNM, a significantly positive relationship with ALDH1A1 expression presented in the America-Europe, NA, and BD subgroups. In addition, the positive relationship between ALDH1A1 and HER2 expression was observed in the cutoff >0/1%, America-Europe, NA, and BD subgroups.

Only one eligible study from Yoshioka *et al*. indicated that ALDH1A1 expression was significantly correlated with larger tumor size (>2.0 cm) [32]. However, our results revealed that high expression of ALDH1A1 correlated with larger tumor size, especially in the cutoff >0/1%, Asia, NA, and BD subgroups. Multicenter prospective studies based on large, homogeneous patient populations will be required to assess the relationship between tumor size and ALDH1A1 expression.

None of the studies eligible for the meta-analysis indicated that ALDH1A1 expression was correlated with LNM. However, our results from larger samples revealed that there is a significant positive association between these two parameters, especially in the America-Europe, NA, and BD subgroups. This is supported by another study by Neumeister *et al*. that was not included in our meta-analysis due to the lack of some required informations. The study indicated that there is a significant association between ALDH1A1 and LNM (OR = 2.37; 95% CI = 1.582–3.165) [19]. In addition, a significant correlation between ALDH1A1 expression in the primary tumor and in the corresponding metastatic lymph nodes has been observed. In a group of 48 breast cancer samples with LNM, Yu *et al*. found that there were 8 ALDH1A1^+^ samples among the primary cancer tissues and 7 positive samples among the corresponding lymph node tissues. In addition, there were 40 ALDH1A1^-^ samples among the primary cancer tissues, and 39 negative cases among the corresponding lymph node tissues (*P* < 0.05) [23]. Similar results were also observed by Nogami [21]. These results suggest that ALDH1A1 might have an important role in LNM, and this relationship was manifested in the results of our meta-analysis. However, there was no significant correlation found between ALDH1A1 expression and LNM in the Asia, ≥stage II, and Abcam subgroups. This indicated that the previous controversial conclusions about ALDH1A1 expression and LNM might result from the different races, clinical stages, and antibodies used in studies; however, there are only 2 studies using the antibody from Abcam, which might reduce the power and accuracy of subcategory analysis. In addition, there is no significant correlation between ALDH1A1 expression and the 5 clinicopathological parameters (tumor size, LNM, SBR grade, PR, and HER2) in the ≥ stage II subgroup. The small number of included studies might also be the reason for this situation. At the same time, it suggests that using the expression level of a single molecule to assess the disease development of advanced breast cancer patients might be inadequate.

Based on the expression patterns of different molecular markers, breast cancer can be divided into more than six similar subgroups, which have distinguishing features with respect to clinical outcomes, responses to adjuvant therapy, and patterns of metastatic recurrence [33,34]. In addition, a recent study suggested that there is a close relationship between the subtypes defined by gene expression profiling and the cellular origin of breast cancer [35,36]. Thus, we also want to know the relationship between ALDH1A1 expression and the three most important molecular markers of breast cancer, ER, PR, and HER2. The results derived from overall analysis suggested that the overexpression of ALDH1A1 might be related to the enriched-HER2 subtype of breast cancer (ER^-^PR^-^HER2^+^), which is derived from the transformation of mammary late luminal progenitor cells [35,36]. However, it should be noted that: First, the positive correlation between ALDH1A1 expression and HER2 is only observed in the America-Europe subgroup. Second, there were discrepancies regarding the definition of HER2 positivity in the different studies. In some studies, tumors with scores of 2+ and 3+ were considered to be HER2 positive (more than 10% of the cells showed positive immunohistochemical staining) [11,23,27]. In other studies, only tumors with scores of 3+ were considered HER2 positive (more than 30% of the cells showed positive immunohistochemical staining) [21,22,26,32]. Only three studies confirmed the amplification of HER2 by fluorescence in-situ hybridization analysis [22,26,29]. Thus, other subtypes defined by gene expression profiling, such as basal-like breast cancer with moderate expression of HER2 (2 + ~3+), might have been included in the HER2^+^ group in this meta-analysis. ALDH1A1 expression might also be related to some basal-like breast cancers, which are derived from the transformation of mammary luminal progenitor cells [35,36]. The results of Nalwoga et al. confirmed this possibility. They found that there was a close relationship between ALDH1A1 expression and the HER2 subtype (OR = 3.6, 95% CI = 1.4–9.7) and the basal-like subtype (OR = 4.0, 95% CI = 1.8–8.8) [28]. Similar results were found in the study presented by Lee [26]. These data suggest that ALDH1A1 could be used as a potential therapeutic target for breast cancers of the HER2-enriched subtype or partial basal-like subtype, especially in patients derived from America-Europe.

It should be noted that there are some limitations to this meta-analysis. First, although we endeavored to extract valid data from survival curves, in which HRs were not directly measured, these indirect data are less reliable than direct data from the original literature because these calculated HRs are the result of univariate analyses and might contain some deviations. Second, all of the studies included in our meta-analyses are retrospective. Their experimental design may contribute to the heterogeneity, which might reduce the analysis power to some extent. Therefore, larger multicenter prospective studies based on homogeneous populations are required to validate the prognostic power of ALDH1A1. Third, publication bias is a concern. We tried to identify all relevant data, but some data were still missing. Some missing information, such as the results presented by Marcato *et al*. [16], might reduce the power of ALDH1A1 as a prognostic predictor in breast cancer patients.

## Conclusion

This meta-analysis indicates that ALDH1A1 is an important predictor of the progression and poor survival of breast cancer patients. Our results suggest that the analysis of ALDH1A1 expression in breast cancer not only provides a better understanding of the relationship between breast tumorigenesis and cancer genomics but may also be beneficial for the design of treatment and the assessment of the prognosis of patients. We will further study the influence of ALDH1A1 expression on differentiation, invasion, and metastasis of breast cancer cells.

## Abbreviations

ALDH1A1: Aldehyde dehydrogenase 1 family member A1; HER2: Epidermal growth factor receptor 2; CSC: Cancer stem cell; ALDH1: Aldehyde dehydrogenase 1; LNM: Lymph node metastasis; ER: Estrogen receptor; PR: Progesterone receptor; OS: Overall survival; DFS: Disease-free survival; HR: Hazard ratio; OR: Odd ratio; SS: Specific survival; RS: Relative survival; MFS: Metastasis-free survival; RFS: Recurrence-free survival; IHC: Immunohistochemistry; IBC: Inflammatory breast cancer; TMA: Tissue microarrays; AQUA: Automated quantitative analysis; TBNC: Triple-negative breast cancer; ALNM: Axillary lymph node metastases; NA: No assessment; NS: No screened.

## Competing interests

The authors declare no conflict of interest.

## Authors’ contributions

YL and DL helped to design the overall study, compile and curate the datasets, design the statistical approaches, perform the computational analysis, and develop the biological interpretation. YL and DL contributed equally to this work. JD and SX provided expertise in clinical breast oncology. JZ and XY helped to design the statistical approaches and perform the computational analysis. YC and XZ helped to design the overall study and design the statistical approaches. SY and XB designed the overall study, compiled and curated the datasets, designed the statistical approaches, performed the computational analysis, developed biological interpretation, and wrote the manuscript. All authors contributed to the preparation of the manuscript and read and approved the final version.

## Pre-publication history

The pre-publication history for this paper can be accessed here:

http://www.biomedcentral.com/1471-2407/14/444/prepub

## Supplementary Material

Additional file 1: Figure S1 Meta-analysis of the association between ALDH1A1 expression and clinicopathological parameters according to the cutoff value of ALDH1A1 expression: (**A**) LNM; (**B**) histological grade; (**C**) tumor size; (**D**) the expression of ER; (**E**) the expression of PR; (**F**) the expression of HER2. Click here for file

Additional file 2: Figure S2 Meta-analysis of the association between ALDH1A1 expression and clinicopathological parameters according to the regions of origin of patients: (**A**) LNM; (**B**) histological grade; (**C**) tumor size; (**D**) the expression of ER; (**E**) the expression of PR; (**F**) the expression of HER2. Click here for file

Additional file 3: Figure S3 Meta-analysis of the association between ALDH1A1 expression and clinicopathological parameters according to the stage of patients: (**A**) LNM; (**B**) histological grade; (**C**) tumor size; (**D**) the expression of ER; (**E**) the expression of PR; (**F**) the expression of HER2. Click here for file

Additional file 4: Figure S4 Meta-analysis of the association between ALDH1A1 expression and clinicopathological parameters according to the different antibodies used in the studies: (**A**) LNM; (**B**) histological grade; (**C**) tumor size; (**D**) the expression of ER; (**E**) the expression of PR; (**F**) the expression of HER2. Click here for file

Additional file 5: Figure S5 Meta-analysis of the association between ALDH1A1 expression and the prognosis according to the regions of origin of patients: (**A**) OS/SS/RS; (**B**) DFS/MFS/RFS. Click here for file

Additional file 6: Figure S6 Meta-analysis of the association between ALDH1A1 expression and the prognosis according to the stage of patients: (**A**) OS/SS/RS; (**B**) DFS/MFS/RFS. Click here for file

Additional file 7: Figure S7 Meta-analysis of the association between ALDH1A1 expression and the prognosis according to the different antibodies used in the studies (DFS/MFS/RFS). Click here for file

Additional file 8: Figure S8 Meta-analysis of the association between ALDH1A1 expression and the prognosis according to the surgery situation of patients: (**A**) OS/SS/RS; (**B**) DFS/MFS/RFS. Click here for file

Additional file 9: Figure S9 Meta-analysis of the association between ALDH1A1 expression and the prognosis according to the cutoff value of ALDH1A1 expression: (**A**) OS/SS/RS; (**B**) DFS/MFS/RFS. Click here for file
