# Supplementary figures and images for: ALDH1A1 expression correlates with clinicopathologic features and poor prognosis of breast cancer patients: a systematic review and meta-analysis
Source: BMC Cancer. 2014 Jun 17;14:444. doi: 10.1186/1471-2407-14-444 (PMC4070403; doi:10.1186/1471-2407-14-444)

## Slide 1
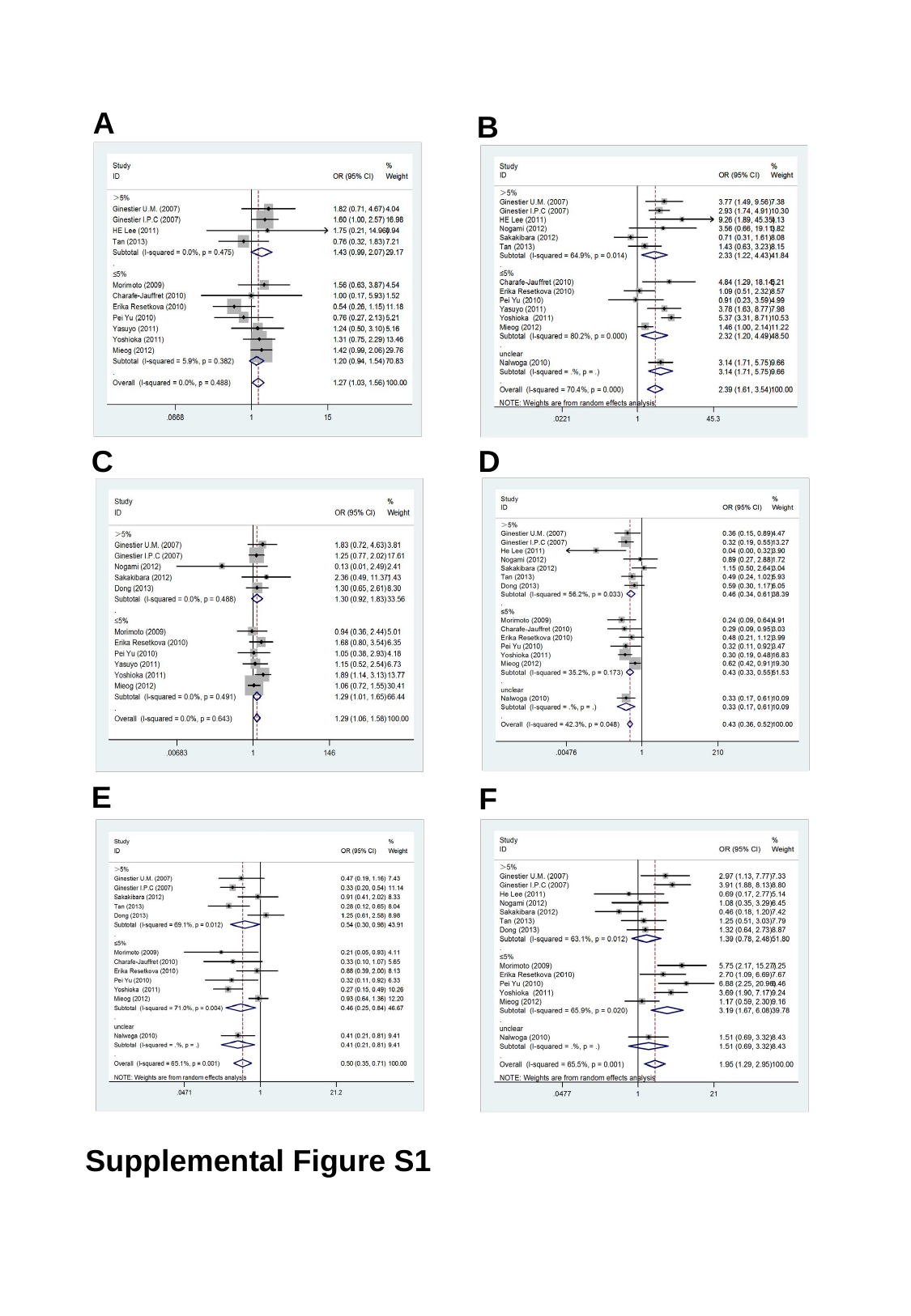

A
B
D
C
E
F
Supplemental Figure S1

Supplement: Additional file 1: Figure S1 — Meta-analysis of the association between ALDH1A1 expression and clinicopathological parameters according to the cutoff value of ALDH1A1 expression: (A) LNM; (B) histological grade; (C) tumor size; (D) the expression of ER; (E) the expression of PR; (F) the expression of HER2. [file 1471-2407-14-444-S1.ppt]

## Slide 1
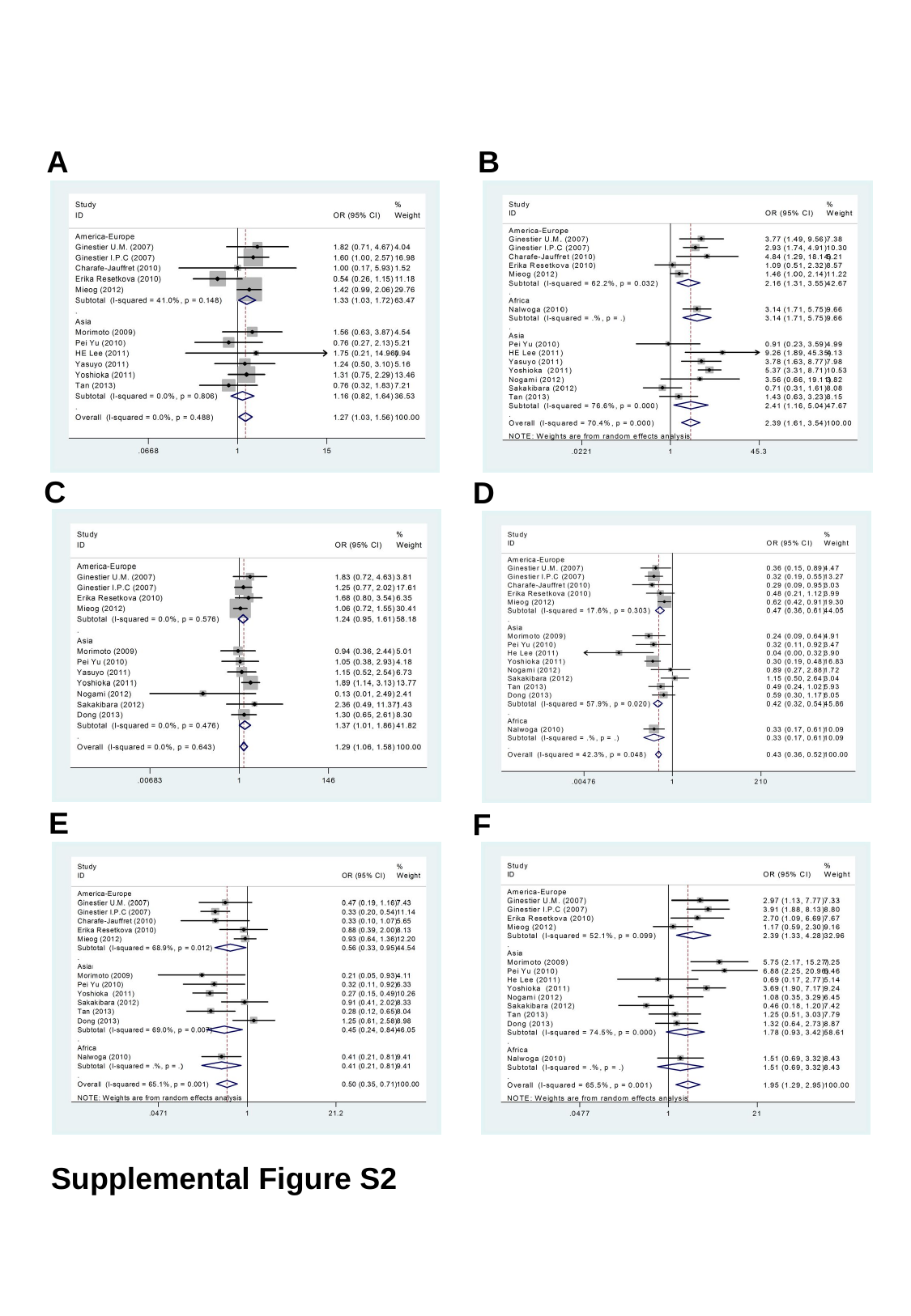

A
B
C
D
E
F
Supplemental Figure S2

Supplement: Additional file 2: Figure S2 — Meta-analysis of the association between ALDH1A1 expression and clinicopathological parameters according to the regions of origin of patients: (A) LNM; (B) histological grade; (C) tumor size; (D) the expression of ER; (E) the expression of PR; (F) the expression of HER2. [file 1471-2407-14-444-S2.ppt]

## Slide 1
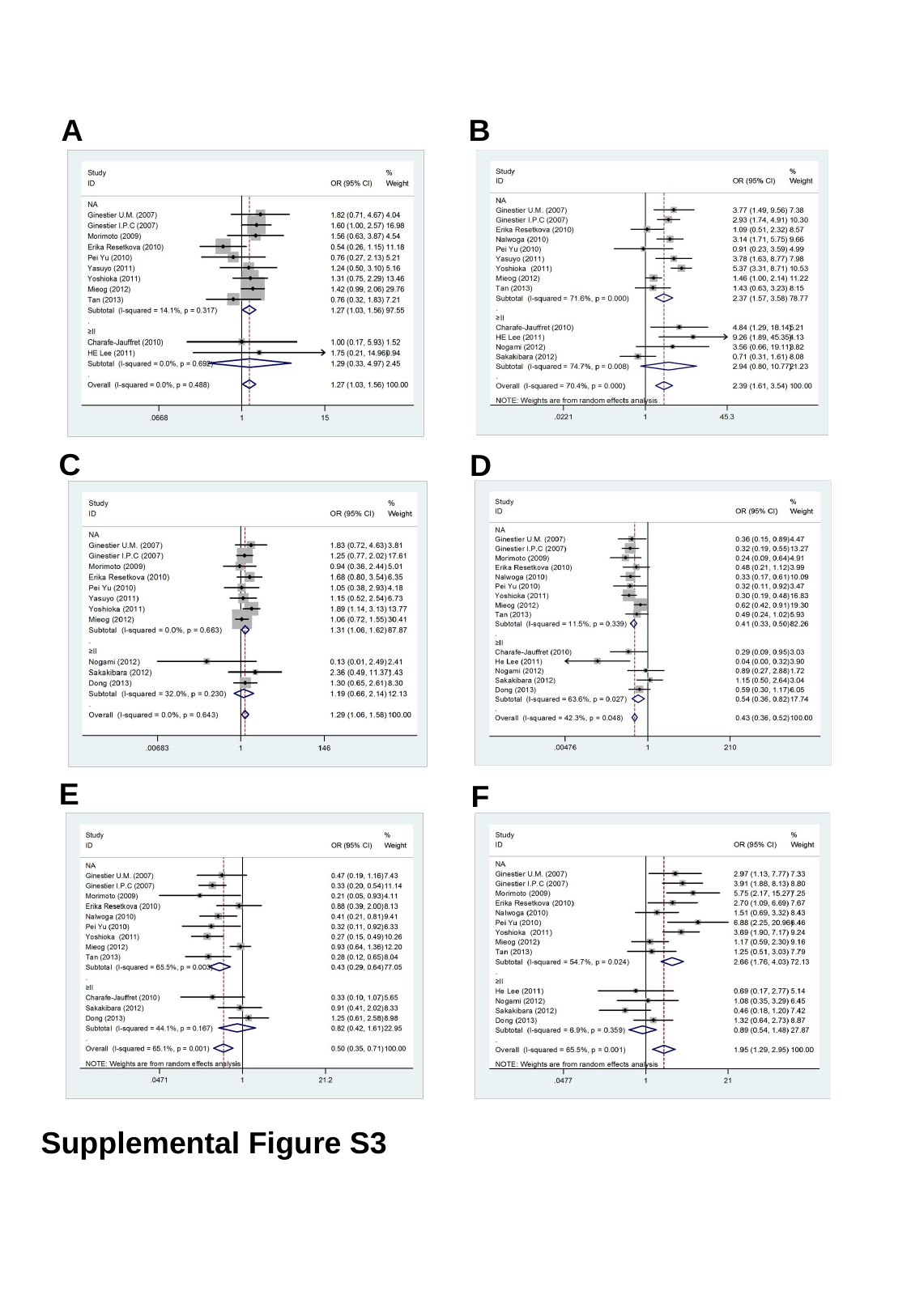

A
B
C
D
E
F
Supplemental Figure S3

Supplement: Additional file 3: Figure S3 — Meta-analysis of the association between ALDH1A1 expression and clinicopathological parameters according to the stage of patients: (A) LNM; (B) histological grade; (C) tumor size; (D) the expression of ER; (E) the expression of PR; (F) the expression of HER2. [file 1471-2407-14-444-S3.ppt]

## Slide 1
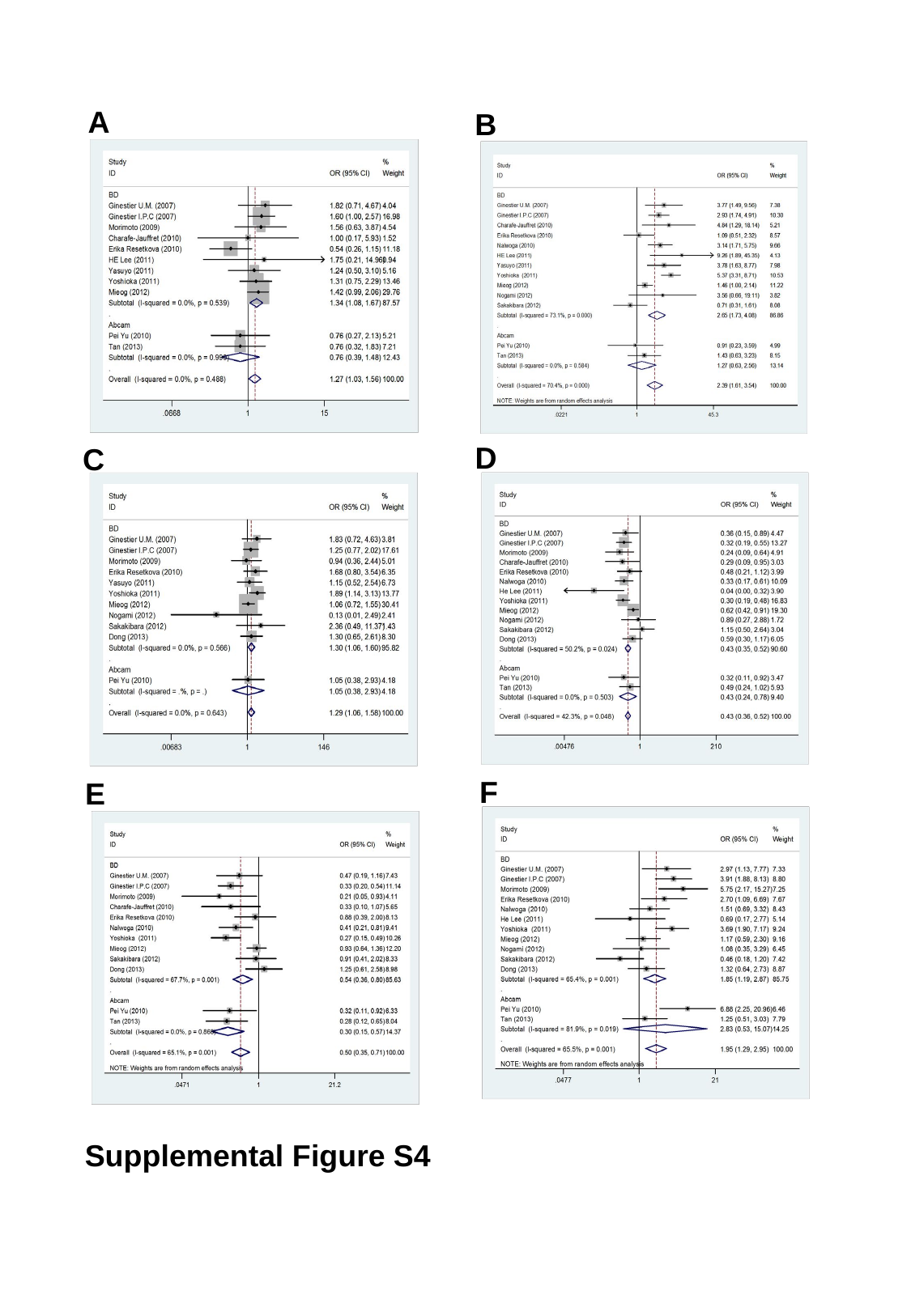

A
B
D
C
F
E
Supplemental Figure S4

Supplement: Additional file 4: Figure S4 — Meta-analysis of the association between ALDH1A1 expression and clinicopathological parameters according to the different antibodies used in the studies: (A) LNM; (B) histological grade; (C) tumor size; (D) the expression of ER; (E) the expression of PR; (F) the expression of HER2. [file 1471-2407-14-444-S4.ppt]

## Slide 1
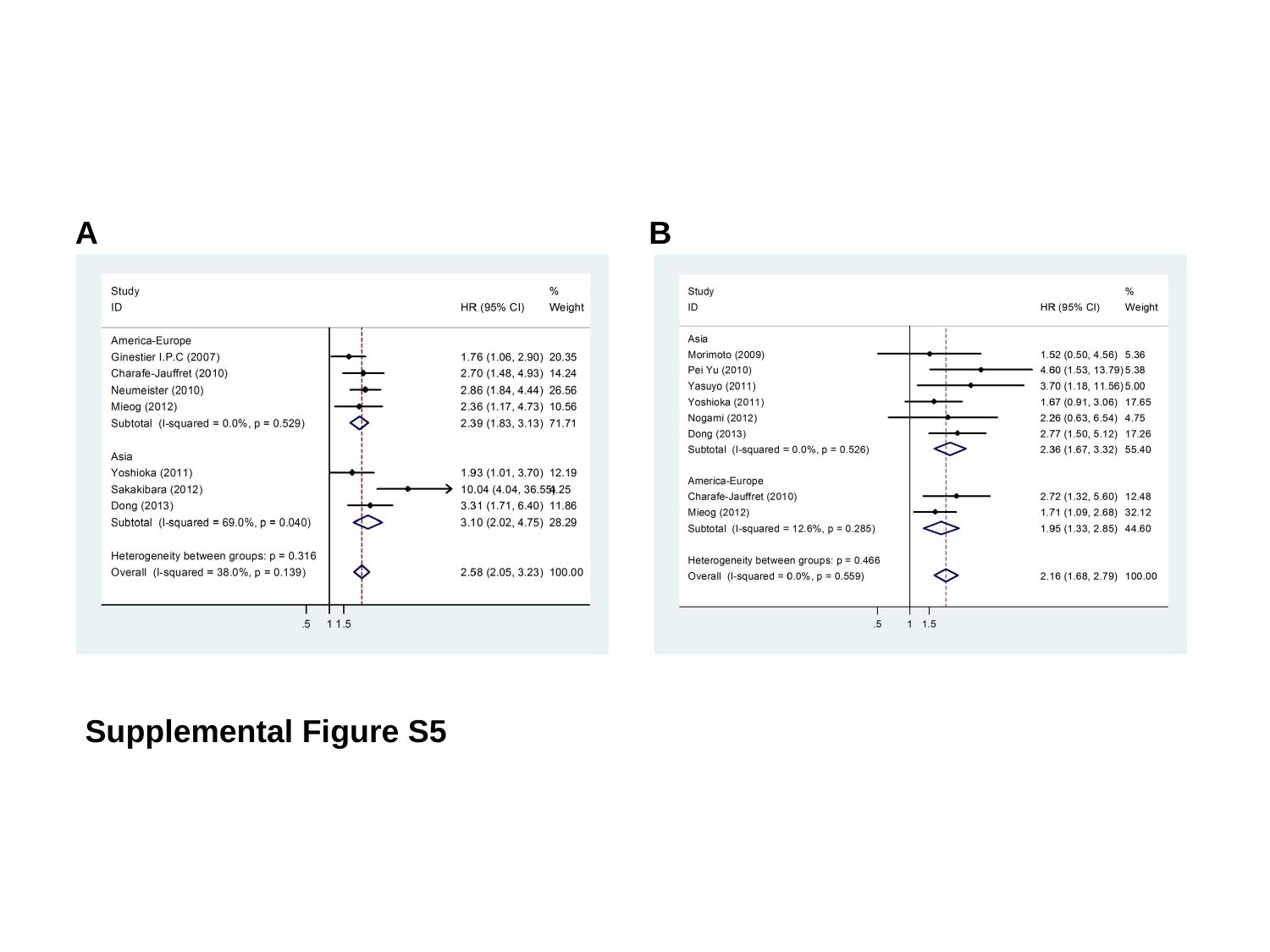

A
B
Supplemental Figure S5

Supplement: Additional file 5: Figure S5 — Meta-analysis of the association between ALDH1A1 expression and the prognosis according to the regions of origin of patients: (A) OS/SS/RS; (B) DFS/MFS/RFS. [file 1471-2407-14-444-S5.ppt]

## Slide 1
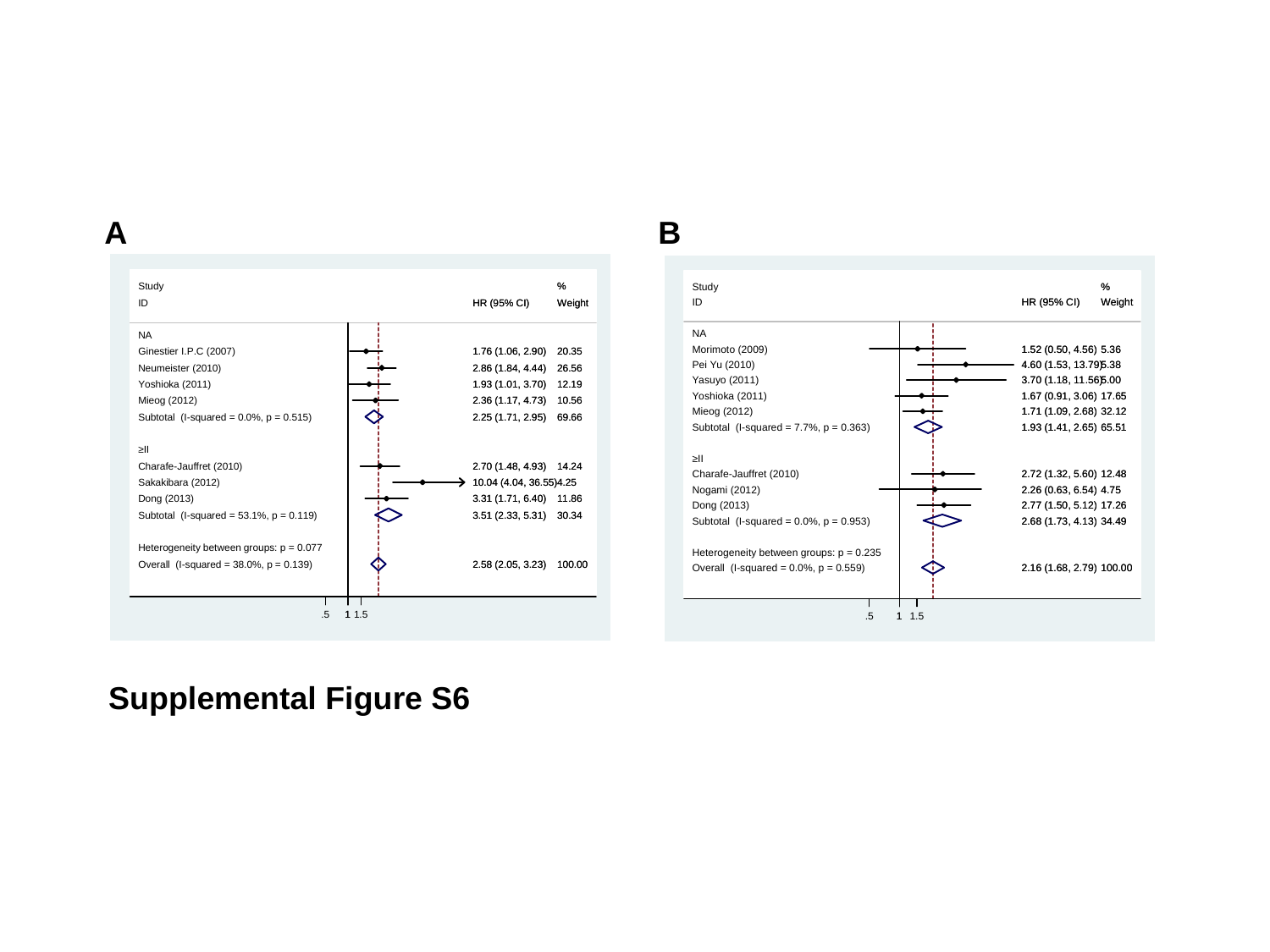

A
B
Supplemental Figure S6

Supplement: Additional file 6: Figure S6 — Meta-analysis of the association between ALDH1A1 expression and the prognosis according to the stage of patients: (A) OS/SS/RS; (B) DFS/MFS/RFS. [file 1471-2407-14-444-S6.ppt]

## Slide 1
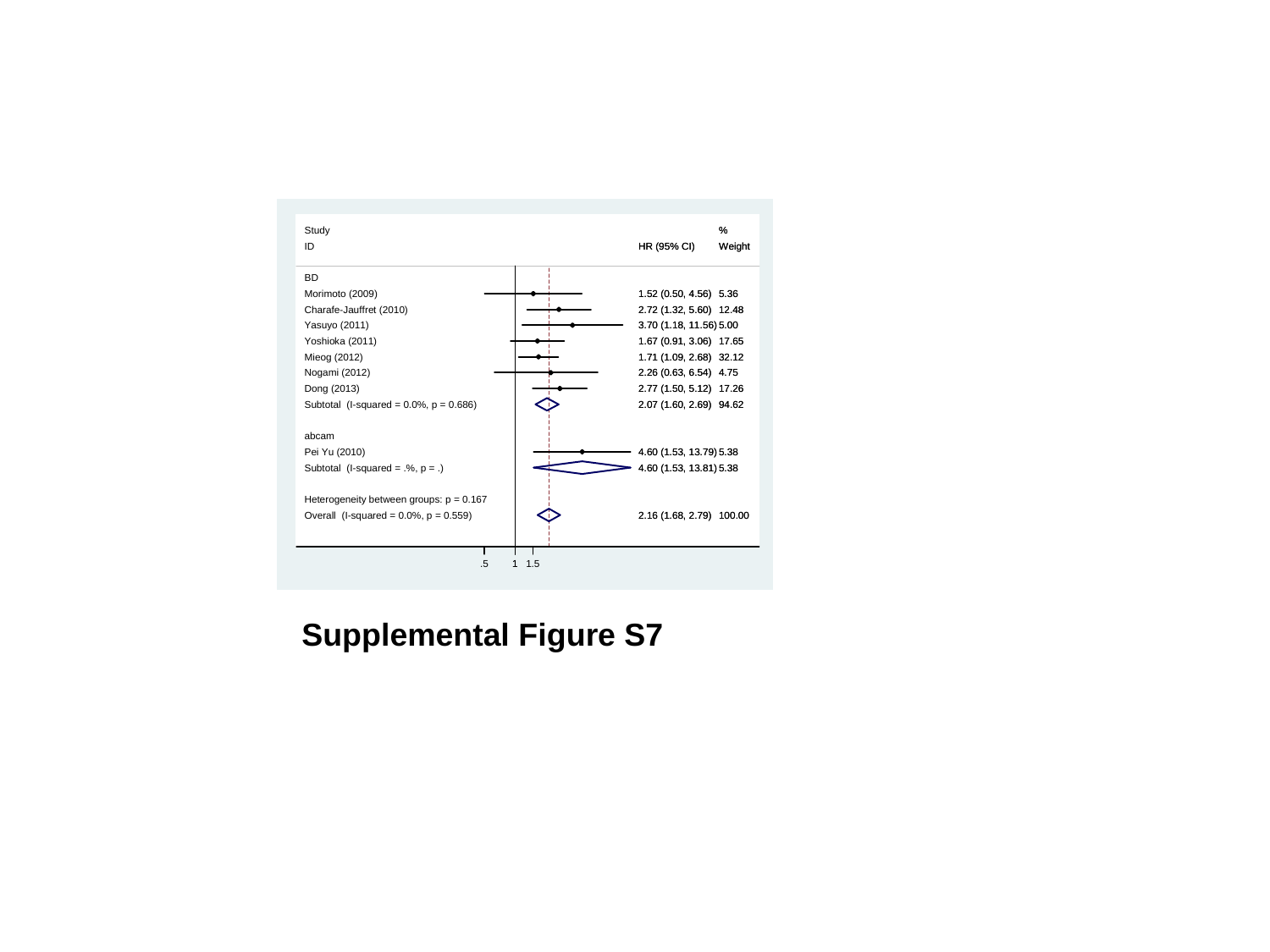

Supplemental Figure S7

Supplement: Additional file 7: Figure S7 — Meta-analysis of the association between ALDH1A1 expression and the prognosis according to the different antibodies used in the studies (DFS/MFS/RFS). [file 1471-2407-14-444-S7.ppt]

## Slide 1
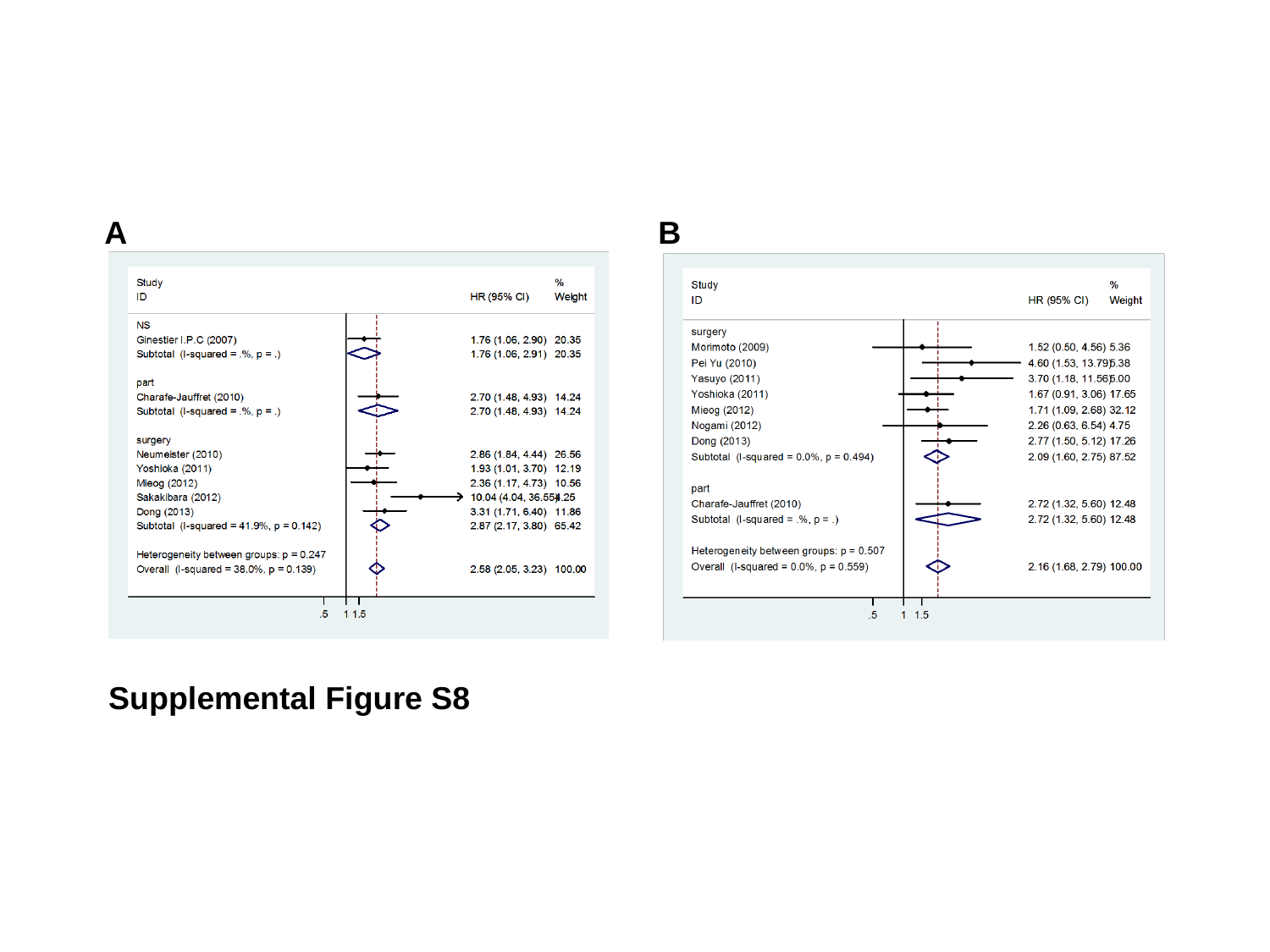

A
B
Supplemental Figure S8

Supplement: Additional file 8: Figure S8 — Meta-analysis of the association between ALDH1A1 expression and the prognosis according to the surgery situation of patients: (A) OS/SS/RS; (B) DFS/MFS/RFS. [file 1471-2407-14-444-S8.ppt]

## Slide 1
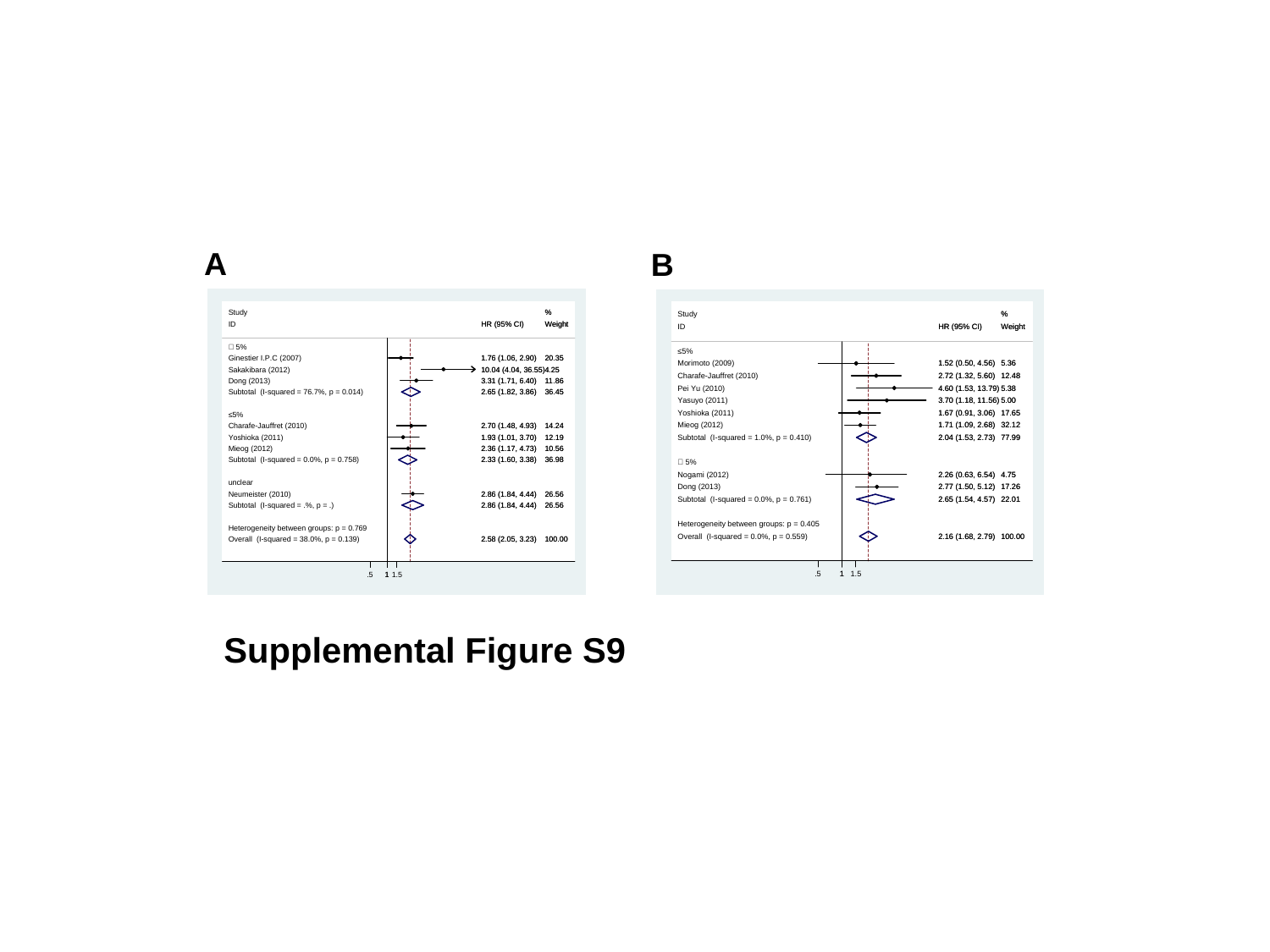

A
B
Supplemental Figure S9

Supplement: Additional file 9: Figure S9 — Meta-analysis of the association between ALDH1A1 expression and the prognosis according to the cutoff value of ALDH1A1 expression: (A) OS/SS/RS; (B) DFS/MFS/RFS. [file 1471-2407-14-444-S9.ppt]
